# A Novel Embeddable Tubular Piezoceramics-Based Smart Aggregate for Damage Detection in Two-Dimensional Concrete Structures

**DOI:** 10.3390/s19071501

**Published:** 2019-03-28

**Authors:** Weihang Gao, Hongnan Li, Siu Chun Michael Ho

**Affiliations:** 1 State Key Lab of Coastal and Offshore Engineering, Dalian University of Technology, Dalian 116024, China; 625922881@mail.dlut.edu.cn; 2 Department of Mechanical Engineering, University of Houston, Houston, TX 77204, USA; smho@uh.edu

**Keywords:** piezoceramic, tubular smart aggregate (TSA), structure health monitoring, two-dimensional (2D) concrete structures

## Abstract

Due to their multiple advantages, piezoceramic materials have been widely used in structural health monitoring (SHM). Piezoceramic patch-based smart aggregate (SA) and spherical piezoceramic-based smart aggregate (SSA) have been developed for damage detection of concrete structures. However, the stress waves generated by these two types of transducers are limited by their geometry and are unsuitable for use in two-dimensional concrete structures (e.g., shear walls, floors and cement concrete pavements). In this paper, a novel embeddable tubular smart aggregate (TSA) based on a piezoceramic tube was designed, fabricated and tested for use in two-dimensional (2D) structures. Due to its special geometry, radially uniform stress waves can be generated, and thus the TSA is suitable for damage detection in planar structures. The suitability of the transducer for use in structural health monitoring was investigated by characterizing the ability of the transducer to transmit and measure stress waves. Three experiments, including impedance analysis, time of arrival analysis and sweep frequency analysis, were conducted to test the proposed TSA. The experimental results show that the proposed TSA is suitable for monitoring the health condition of two-dimensional concrete structures.

## 1. Introduction

Structural health monitoring [1,2,3] and damage detection [4,5,6] techniques have demonstrated their critical roles in detecting potential hazards in civil structures [7,8,9,10], including concrete structures. Over decades of development, many advanced transducers have been reported in the literature to increase the accuracy of damage detection. The most widely reported categories of transducers include piezoelectric transducers [11,12,13,14], optical fiber sensors [15,16,17] and magnetic flux transducers [18], among others.

Piezoelectric-based transducers are commonly used for the structural health monitoring of concrete structures. Due to their rapid response times, low cost and availability in different geometries, piezoelectric transducers are a staple of many practical projects [19,20,21,22,23,24]. Furthermore, owing to the direct and inverse piezoelectric effect of piezoelectric materials, piezoelectric-based transducers can both transmit and receive stress signals [25,26,27,28,29]. Yang and Divsholi [30] proposed a damage detection method based on the electromechanical impedance technique. The large frequency range (30–400 kHz) examined in their research was divided into sub-frequency intervals. The root mean square deviation of each sub-frequency interval was then calculated to estimate the location of the damage. Liang et al. [31] demonstrated the use of piezoceramic transducers to monitor the occurrence of bond-slip in concrete-encased composite structures. In their research, the root mean square deviation is employed to define the damage condition of the bond-slip. The simulation and experimental results revealed that their damage detection method could detect the bond-slip damage. Gao et al. [32] introduced the concept of the smart concrete slab, which is enabled by the embedment of lead zirconate titanate (PZT) transducer arrays. Based on a delay-and-sum imaging algorithm, the damage in the concrete slab could be visualized. However, piezoelectric patches are very vulnerable to external loads, and impacts on the surface may destroy them. The intrusion of conductive media such as water into the concrete can short out and decapacitate the piezoelectric transducers. During a cycle of active sensing, the direct short will increase the risk of shock.

To resolve the aforementioned problems, smart aggregates (SAs) were proposed by Song et al. [33]. In the SA, the PZT patch is protected by an inner metal shell (or hydro insulating material) and an external layer consisting of high-strength protective material (marble or cement). The external protective shell enables the PZT patch to safely function even as the concrete is being poured in the early stages of construction. Furthermore, the SA retains the ability of conventional PZT patches to transmit and receive the stress waves with designed frequencies. A large amount of research has been performed to verify the practicability and the feasibility of SAs in damage detection and structural health monitoring [34,35,36,37,38,39,40]. Feng et al. [41] demonstrated the use of embedded smart aggregates to track the strength development of early-age concrete in real time based on the active sensing approach. Experimental results showed that the hydration monitoring index developed by the authors to quantify the signals exhibited a similar trend to the 28 day concrete strength curve. Yan et al. [42] employed the smart aggregate to achieve the structural health monitoring of a concrete shear wall. Song et al. [43] reported on the use of smart aggregates to detect collisions and evaluate the severity of the collisions for a concrete bridge. However, due to the plate-like geometry of the inner PZT patch, stress waves generated by the conventional SA can be considered as unidimensional. Hence the detection range of the conventional SA is limited to the patch orientation. Therefore, a large number of SAs are needed to adequately monitor a concrete structure.

**Figure 1:**
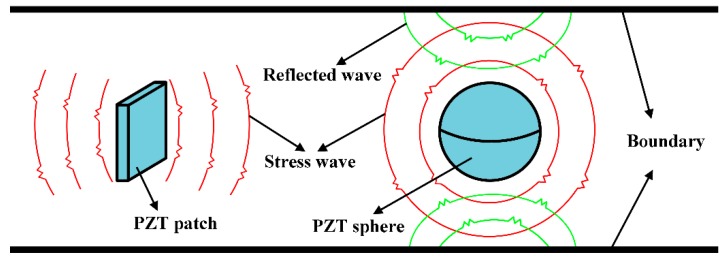
Stress waves generated by a lead zirconate titanate (PZT) patch vs. a PZT sphere in a two-dimensional concrete structure.

More recently, a spherical smart aggregate (SSA) was proposed by Kong et al. [44,45]. In the SSA, the PZT patch in the conventional SA is replaced by a spherical piezoceramic shell. The proposed SSA can offer omni-directional actuating and sensing capabilities and improve the detection aperture for damage detection in concrete structures. However, in practical engineering, many concrete structures can be considered as two-dimensional structures (e.g., precast shear wall, concrete pavements and floors). If the SSA is employed to generate and measure stress waves in two-dimensional (2D) structures, reflected waves from the two surfaces along the direction of the thickness will complicate the detection result. Meanwhile, for high-accuracy and sensitivity damage localization and imaging methods, the reflected waves may become noise. Figure 1 illustrates the stress waves generated by the PZT sphere inside the SSA compared to the stress waves generated by a PZT patch inside the conventional SA in a two-dimensional concrete structure.

To make the shapes of the generated stress wave more compatible with 2D structures, a novel tubular smart aggregate (TSA) was developed in this research. The actuating and sensing core in the TSA is a piezoceramic tube, which can generate a radially expanding stress wave along the plane of slab-like concrete structures while minimizing reflections at the two surfaces. This paper is organized as follows: The fabrication of the TSA is detailed in Section 2. The ability of the TSA to generate and receive stress waves was tested on a hexahedral concrete specimen, as described in Section 3. The experiment results are presented and discussed in Section 4, and the conclusions are drawn in Section 5.

## 2. Fabrication of the Tubular Piezoceramic Smart Aggregate

### 2.1. Electrical Insulation and Waterproofing

**Figure 2:**
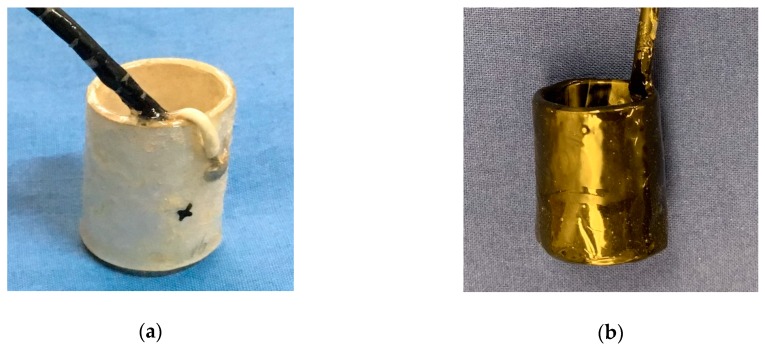
Electrical insulation and waterproofing process: (**a**) epoxy resin protective coating and (**b**) liquid electrical tape protective layer.

The proposed TSA was composed of a piezoceramic tube as the core. The inner diameter, the height and the thickness of the piezoceramic tube were 16 mm, 25 mm and 2 mm, respectively. After soldering the wire electrode, a thin layer of epoxy resin was coated over the piezoceramic tube to enhance the strength of the piezoceramic materials and provide a waterproofing layer. Finally, a liquid electrical tape was coated over the entire assembly to provide electrical insulation and further improve the waterproofing. The entire electrical insulation and waterproofing process is shown in Figure 2.

### 2.2. Electrical Isolation and Waterproofing

Due to the fragility of piezoceramic material, care must be taken during the casting of concrete to prevent damaging the transducers. Therefore, to ensure the durability of the proposed TSA, the piezoceramic tube was encased within a layer of ultra-high-performance concrete (UHPC), which formed a high strength protective layer around the piezoceramic tube. UHPC is a suitable choice due to its high strength compared with normal concrete [44].

**Figure 3:**
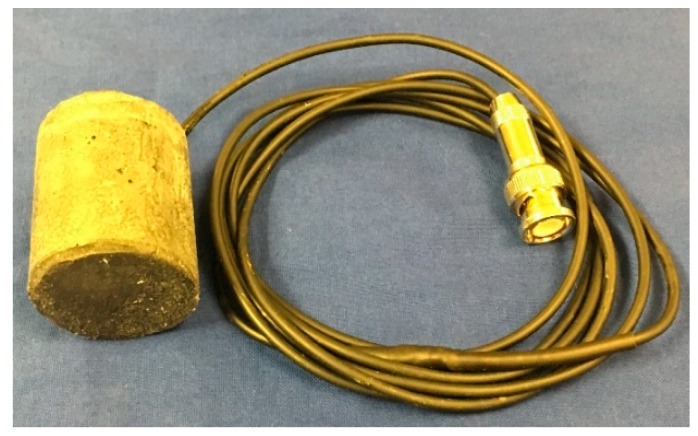
A fabricated tubular smart aggregate (TSA).

To form the UHPC layer, a two-part mold which includes a wooden pedestal and a polyvinyl chloride tube was fabricated. The tube was cut along the length to form two half tubes, which were then bonded together by epoxy resin. Alternatively, if larger scale production is considered, the mold can be fabricated by a three-dimensional (3D) printer. After the mold was prepared, the electrically insulated and waterproofed piezoceramic tube was suspended in the mold by a fine copper wire. After the UHPC was poured into the mold, the completed TSA could be removed from the mold after 28 days of curing. The diameter and the height of the TSA was 25 mm and 40 mm, respectively (Figure 3).

## 3. Experimental Setup

### 3.1. Experimental Specimen

**Figure 4:**
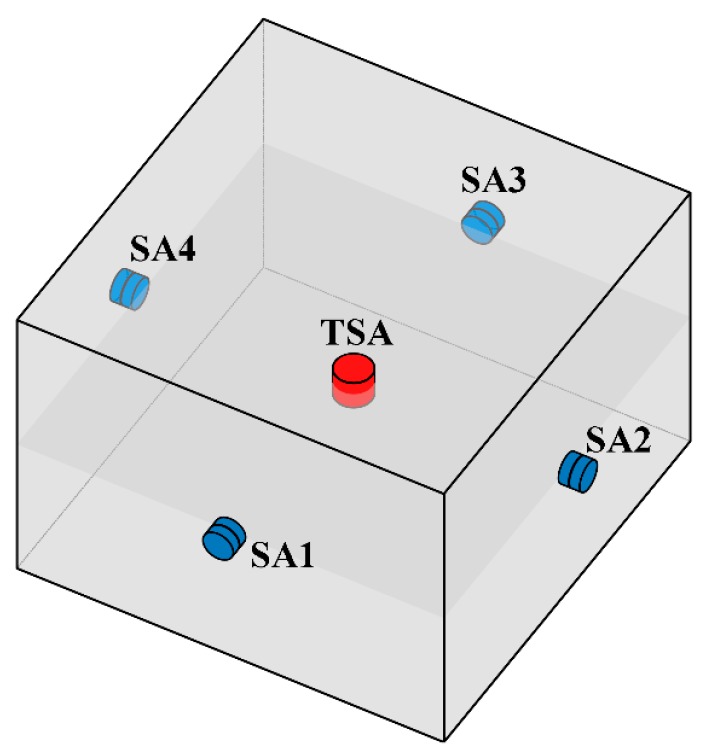
The diagram of the concrete specimen with embedded TSA and surface-bonded smart aggregates (SAs).

The functionality of the TSA was tested by embedding it in a hexahedral concrete specimen. The specimen was 280 mm in length, 280 mm in width, and 200 mm in height. The detailed parameters of the concrete for casting the experimental specimen are listed in Table 1. In order to enable a clearer understanding of the TSA characteristics in generating and receiving stress waves, the TSA was embedded in the center of the specimen. Then, four conventional unidimensional SAs were bonded onto the surface of the concrete specimen. It should be noted that the centroids of the TSA and SAs should be kept evenly in the same horizontal plane. The diagram of the concrete specimen is shown in Figure 4.

### 3.2. Experimental Specimen

**Figure 5:**
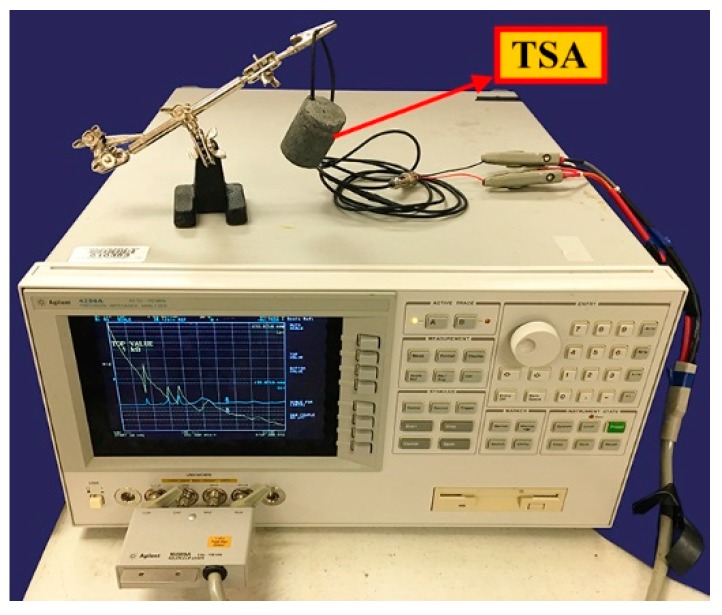
The experimental setup for impedance analysis.

**Figure 6:**
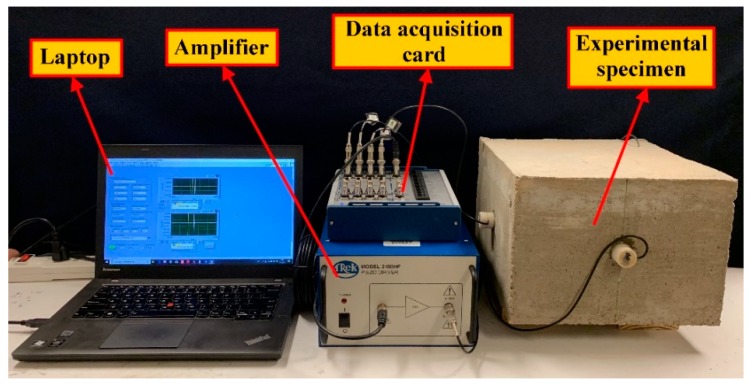
The experimental setup for time of arrival analysis and sweep frequency analysis.

Three experiments were conducted to study the characteristics of the proposed TSA. In the first experiment, an impedance analyzer (Agilent 4294A, Santa Clara, CA, USA) was used to analyze the natural frequencies of the proposed TSA (Figure 5). Based on the measured natural frequencies, suitable operating frequencies of the TSA can be determined. In the second and third experiments, a data acquisition card (NI USB-6361, Austin, TX, USA) was used to generate and receive signals, and a power amplifier with a 150 V output voltage was employed to strengthen the signal input to the TSA and SAs for stress wave generation. The entire experimental setup for the second and third experiments is shown in Figure 6.

The second experiment aimed to demonstrate the advantage of the proposed TSA over conventional SAs [33] and SSAs [44,45] with regards to generating and receiving stress waves in two-dimensional concrete structures. In the third experiment, a swept sine signal was used to study its applicability of the TSA in implementing the commonly used active sensing approach [46]. For the sake of completeness, the proposed TSA was used in turn as an actuator and a sensor in both experiments.

## 4. Results and Discussion

### 4.1. Impedance Analysis of the Proposed TSA

**Figure 7:**
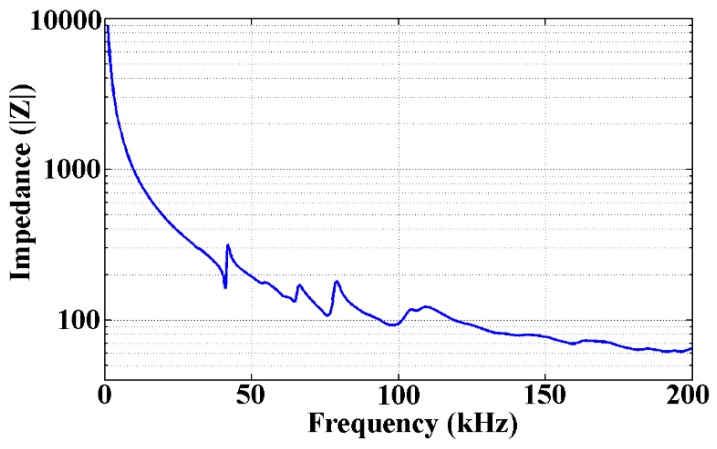
The experimental result of impedance analysis of the TSA.

Impedance analysis was performed to study the natural frequencies of the TSA and the operational frequency ranges. As shown in Figure 5, to ensure free boundary conditions, the test sample was suspended by its connection wire via a clamp. The impedance analysis results are displayed in Figure 7. The first and second natural frequencies are in the ranges of 35–45 kHz and 60–85 kHz, respectively. The higher natural frequencies occur beyond 90 kHz. This result indicates that the suitable operating frequency ranges of this TSA were 35–45 kHz and 60–85 kHz. Stress waves in this frequency range can be transmitted over longer distances.

### 4.2. Time of Arrival Analysis of the Proposed TSA

**Figure 8:**
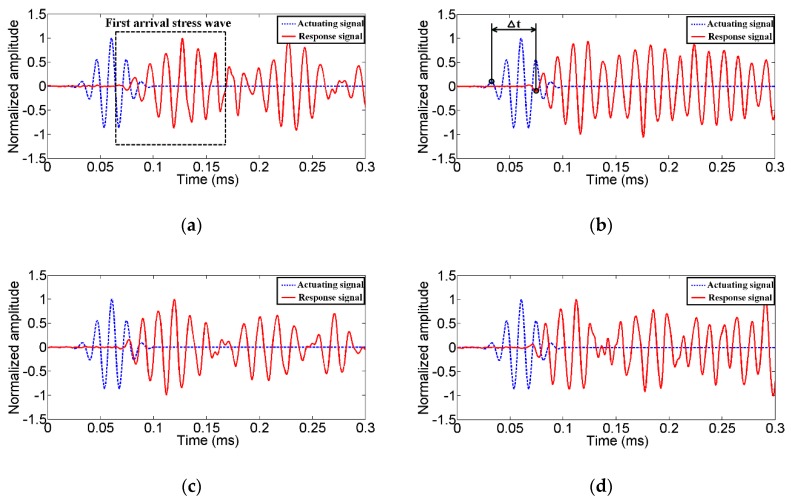
The time of arrival analysis when the TSA works as an actuator and (**a**) SA1 works as a sensor, (**b**) SA2 works as a sensor, (**c**) SA3 works as a sensor, (**d**) SA4 works as a sensor.

**Figure 9:**
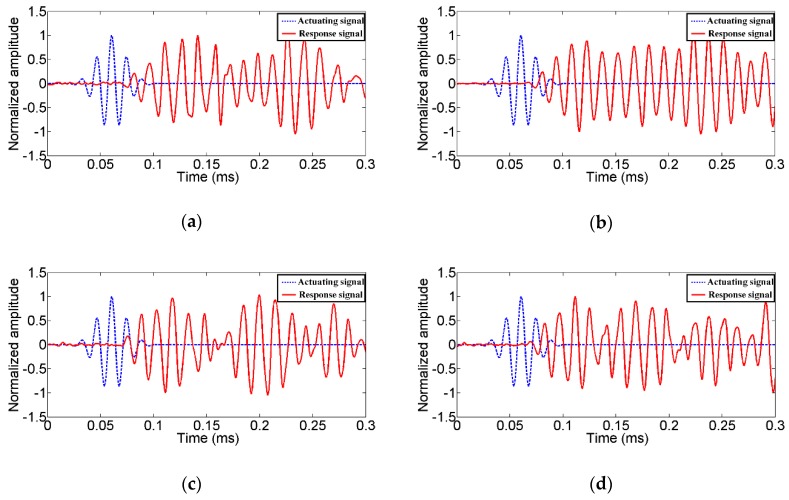
The time of arrival analysis when the TSA works as a sensor and (**a**) SA1 works as an actuator, (**b**) SA2 works as an actuator, (**c**) SA3 works as an actuator, (**d**) SA4 works as an actuator.

To characterize the abilities of the TSA to generate and receive stress waves in 2D structures, a time of arrival analysis was conducted. The results of the second experiment are shown in Figure 8. The broken blue lines in Figure 8 and Figure 9 represent the actuating signals. Guided by the impedance results, a modulated Gaussian pulse with a center frequency of 70 kHz was employed to actuate the transducers [47]. Meanwhile, the solid red lines represent the response signals received by the surface bonded conventional SAs. For the sake of clarity, all the received signals in this experiment were normalized by the intensity of the first arrival stress wave.

From Figure 8 and Figure 9, the advantage of the proposed TSA over the conventional SA and the SSA can be observed. When the TSA serves as the actuator, a radially uniform stress wave in the 2D plane of the structure can be generated. The first arrival of the stress wave received by SAs can be clearly identified. Likewise, the proposed TSA can easily recognize the first arrival of the stress wave when serving as a sensor. Compared to the SA, the TSA has a larger monitoring area. Therefore, fewer transducers are needed. Compared to the SSA, the influence of the reflected stress waves from the surface boundaries in the two-dimensional concrete structure can be minimized. Please note that many damage detection methods, such as those time-of-arrival based damage localization [48,49,50] and imaged based damage detection methods [51,52,53,54], require the proper identification of the first arrival of the stress wave. With the proposed TSA, more accurate time of arrival and therefore more accurate damage detection results can be obtained. In addition, the determination of the location of an impact on a 2D structure also requires the accurate arrival time [55,56,57], which can be provided using the proposed TSA transducers.

According to prior research [32,47], the first arrival of the stress wave in concretes are typically composed of longitudinal waves. Therefore, the velocity of the first arrival stress wave can be computed by Equation (1):

(1) $$v_{LT}=\frac{\Delta L}{\Delta t}$$

where v*_LT_* is the velocity of the longitudinal waves, ∆*L* is the distance between the transmitter and the receiver and ∆*t* denotes the time difference between the actuating signal and the corresponding receiving signal, as shown in Figure 8b.

In this way, the velocity of each test can be computed. The results are listed in Table 2. T-A1 denotes the calculated result of the velocity when the TSA works as an actuator and SA1 works as a sensor. Meanwhile, A1-T denotes the calculated result of the velocity when SA1 works as an actuator and the TSA works as a sensor. The average velocity of this concrete specimen is 3290.6 m/s. The greatest difference between the computed velocity and the average velocity is about 3.77%, which demonstrates that the proposed TSA can accurately obtain the velocity regardless of its role as an actuator or sensor. It should be noticed that the velocity difference may be contributed to by installation errors of the transducers.

### 4.3. Sweep Frequency Analysis of the Proposed TSA

**Figure 10:**
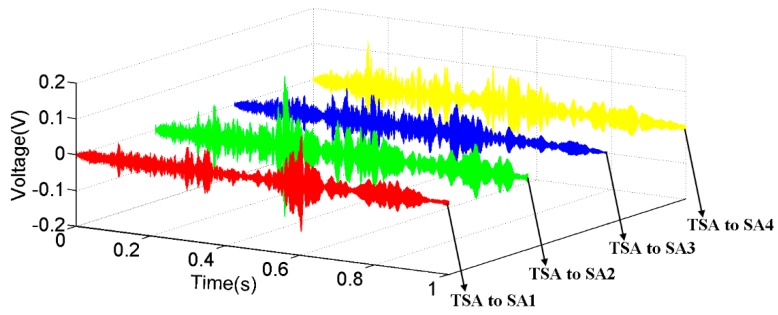
The received signals of the surface bonded SAs when the TSA generated a radially uniform sweep sine excitation.

The received time-domain signal responses of the surface bonded unidimensional SA when using the TSA as the actuator are shown in Figure 10. The experimental results confirm that the stress wave induced by the TSA is radially uniform in the plane of actuation. The signals received by the conventional SA in the four directions are all distinct with high signal to noise ratios (SNRs). This result demonstrates that the TSA is suitable for implementing stress wave-based damage detection methods, such as the active sensing method, for concrete structures. Please note that the piezoceramic-based active sensing methods [58,59] have been widely researched for structural health monitoring and damage detection of many different structures [60,61]. With the high SNR of the proposed TSA for a 2D concrete structure, it is expected that active sensing using the proposed TSAs will have improved damage detection results for such a structure.

**Figure 11:**
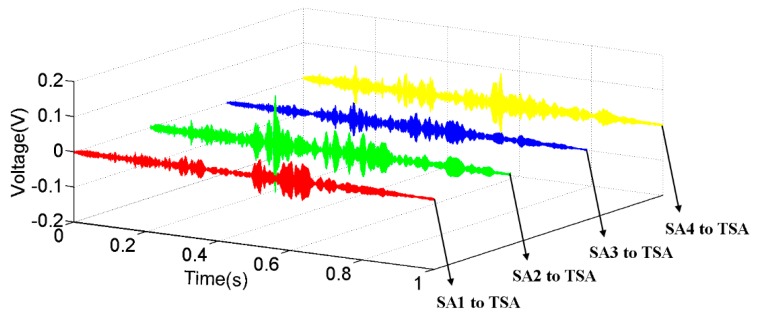
The received signals of the embedded TSA when the surface bonded SAs generated a sweep sine excitation.

Meanwhile, the proposed TSA can also clearly receive signals from all directions within the plane. As shown in Figure 11, when the surface bonded SAs were in turn used to generate sweep sine signals, the TSA can receive the stress waves from all four directions. However, due to the differences in size and thickness between the PZT patch inside the SA and the piezoceramic tube inside the TSA, the stress wave generation capacity is different between the TSA and SAs. Compared to the conventional SA, TSA can generate more intense stress waves. As a result, the SNR was lower when the SA served as the actuator (Figure 10 vs. Figure 11). This result further verifies the ability of the TSA for use in the damage detection of concrete structures. Under the same input voltage, the stress wave induced by the TSA can propagate across a greater distance. In this way, the monitoring area of the proposed TSA is enlarged compared with a similarly fabricated SA.

## 5. Conclusions

In this research, a new type of smart aggregate (SA) using a piezoceramic tube is proposed. Compared to the conventional piezoceramic patch-based SAs and the recently reported piezoceramic SSAs, this tubular smart aggregate (TSA) is more suitable for actuating and receiving stress waves in two-dimensional concrete structures. Three experiments were conducted to characterize the TSA. In the first experiment, the natural frequencies of TSA were analyzed to determine the suitable operating frequencies. The unique feature of the TSA in generating and receiving radially uniform stress waves was tested in the second experiment. Finally, the ability of the TSA to implement active sensing was tested by transmitting and receiving swept sine stress waves with the surrounding SAs. The experimental results indicate the feasibility of the proposed TSA in the damage detection of two-dimensional concrete structures. In the authors’ future work, a numerical simulation will be conducted to further study the characteristics of the proposed TSA, including the optimal height of the TSA needed to induce the appropriate stress waves in two-dimensional concrete structures and the optimal thickness of the protective layer outside the TSA. In addition, the performance of the TSA for different applications of damage detection and structural health monitoring will be investigated.

## Figures and Tables

**Table 1:** The detailed parameters of the concrete.

Water (kg/m^−3^)	Cement (kg/m^−3^)	Sand (kg/m^−3^)	Gravel (kg/m^−3^)
190	390	755	1090

**Table 2:** The velocity computation result.

	T-A1	T-A2	T-A3	T-A4	A1-T	A2-T	A3-T	A4-T	Average
Velocity (m/s)	3333.3	3333.3	3218.4	3218.4	3294.1	3414.6	3294.1	3218.4	3290.6
Error (%)	1.30	1.30	−2.19	−2.19	0.11	3.77	0.11	−2.19	
